# Diagnostic value of serum human epididymis protein 4 and cancer antigen 125 in the patients with ovarian carcinoma: A protocol for systematic review and meta-analysis

**DOI:** 10.1097/MD.0000000000025981

**Published:** 2021-05-28

**Authors:** Hai-Ying Dai, Fang Hu, Yuan Ding

**Affiliations:** a Department of Clinical Laboratory, Huangshi Central Hospital (Affiliated Hospital of Hubei Polytechnic University), Edong Healthcare Group, Huangshi; b Department of Clinical Laboratory, Hanchuan People's Hospital, Hanchuan, Hubei Province, China.

**Keywords:** cancer antigen 125, diagnostic, human epididymis protein 4, meta-analysis, ovarian carcinoma

## Abstract

**Background:**

Ovarian carcinoma (OC) is considered among the most prevalent triggers of cancer-related deaths in women. Many studies have demonstrated that human epididymis protein 4 (HE-4) as well as cancer antigen 125 (CA-125) are over-expressed in various malignant tumors, such as lung, liver, endometrial, gastric, breast, as well as ovarian cancers. Nonetheless, the overall diagnostic value of serum HE-4, in addition to CA-125 n patients experiencing OC, is still largely undetermined. Therefore, the current study intends to investigate the general diagnostic significance of HE-4 along with CA-125 in patients with OC.

**Methods:**

We aim to systematically search retrospective or prospective study for potential eligible studies from electronic databases, such as MEDLINE, EMBASE, Cochrane Library, Web of Science, as well as Chinese National Knowledge Infrastructure. We will relevant articles evaluating the general diagnostic significance of HE-4 and CA-125 in patients with OC from these databases. We will define our search in English and Chinese. Likewise, we will use 2 independent authors to extract the required data, using the Quality Assessment of Diagnostic Accuracy Studies-2 tool to evaluate he procedural quality of all included literature. We will use the appropriate statistical method to complete data analyses.

**Results:**

The present study aims to investigate the general diagnostic significance of HE-4 and CA-125 in patients suffering from OC.

**Conclusion:**

The present study will systematically summarise current evidence of HE-4 in combination with CA-125 in relation to diagnosing OC.

**Ethics and dissemination:**

Ethical approval will not be required.

**Protocol registration number:**

DOI 10.17605/OSF.IO/YQPC7 (https://osf.io/yqpc7/).

## 1 Introduction

According to statistics, ovarian carcinoma (OC) is considered the seventh primary cause of all cancer-related deaths among women worldwide, accounting for approximately 4.7% of all cancer mortality among women and one of the deadliest gynecological cancers.^[1,2]^ Based on the GLOBOCAN estimates, an estimated 313,959 women were diagnosed with OC in 2020, and nearly 207,252 deaths resulting from the disease.^[1]^ Because OC lacks specific early symptoms, primarily in early-stage (I, II) OC, many patients are usually at the late stages (III, IV) when diagnosed, with a probability of five-year survival rate of about 47%, which drops sharply to approximately 20% in stage IV.^[3,4]^ Regardless of the signs of progress in the identification and treatment of OC over the past decades, the outcomes of OC patients are still unsatisfactory.

Such serum molecular biomarkers employed to diagnose and follow-up patients suffering from OC are carbohydrate antigen 199, carcinoembryonic antigen, fetal alpha protein, human epididymis protein 4 (HE-4), and cancer antigen 125 (CA-125).^[5,6]^ Additionally, they can be utilized to monitor tumor relapse or progression. In particular, they have been used considerably tumor recurrence or progression in patient management. Still, clinical usage of these markers has been restricted due to the lack of sensitivity. Currently, CA-125 and HE-4 are well-established molecular biomarkers in OC diagnosis.^[7]^ Many studies have demonstrated that HE-4 is a better OC molecular biomarker compared to CA-125. HE-4 is augmented in an estimated 90% of women patients experiencing OC.^[8–10]^ Besides, HE-4 has an advanced specificity compared to CA-125 regarding differentiating malignant and benign gynecologic disease.^[11,12]^ Still, the overall diagnostic significance of serum HE-4 and CA-125 in patients suffering from OC is essentially indefinite. Therefore, the present study will explore the overall diagnostic value of HE-4 in combination with CA-125 in patients with OC.

## 2 Objectives

This protocol seeks to examine the general diagnostic significance of HE-4 in combination with CA-125 in patients with OC.

## 3 Methods

### 3.1 Study registration and design

The protocol has been registered on the Open Science Framework (OSF, http://osf.io/) with a registration number 10.17605/OSF.IO/YQPC7. It will be designed using guidelines put forward by the Preferred Reporting Items for Systematic Review and Meta-Analysis Protocol (PRISMA-P) statement.^[13]^

## 4 Eligibility criteria for included studies

### 4.1 Type of studies

The study will consider a retrospective or prospective investigation of the overall diagnostic value of HE-4 and CA-125 among patients experiencing OC.

### 4.2 Type of participants

We will include participants who were diagnosed with OC and confirmed by histopathology.

### 4.3 Type of index test

Blood-based specimens (the expressions of HE-4 and CA-125 will be detected via immunohistochemistry). Likewise, serum-based specimens (the levels of HE-4 and CA-125 will be detected via enzyme-linked immunosorbent assay or chemiluminescent microparticle immunoassay).

### 4.4 Type of outcome measures

The outcome measures include diagnosis odds ratio, positive and negative likelihood ratios, the area under the curve, sensitivity, specificity, summary receiver operating characteristic, and their 95% confidence intervals.

## 5 Data sources and search strategy

We will systematically search retrospective or prospective studies for potentially eligible studies from MEDLINE, EMBASE, Cochrane Library, Web of Science, and Chinese National Knowledge Infrastructure databases. We will collect articles from these electronic databases and use English and Chinese languages. This will help to evaluate the general diagnostic significance of HE-4 and CA-125 among patients experiencing OC. We will use the medical subject heading terms and full-text words for the search. Some of the terms include the following: “human epididymis protein 4,” “HE-4,” “human epididymis 4,” “human epididymis secretory protein 4,” “HE4 protein,” “cancer antigen 125,” “CA-125,” “carbohydrate antigen 125,” “ovarian cancer,” “ovarian carcinoma,” “ovarian tumour,” “ovarian tumour,” and “ovarian neoplasm.”

## 6 Data collection and analysis

### 6.1 Study selection

**Figure 1:**
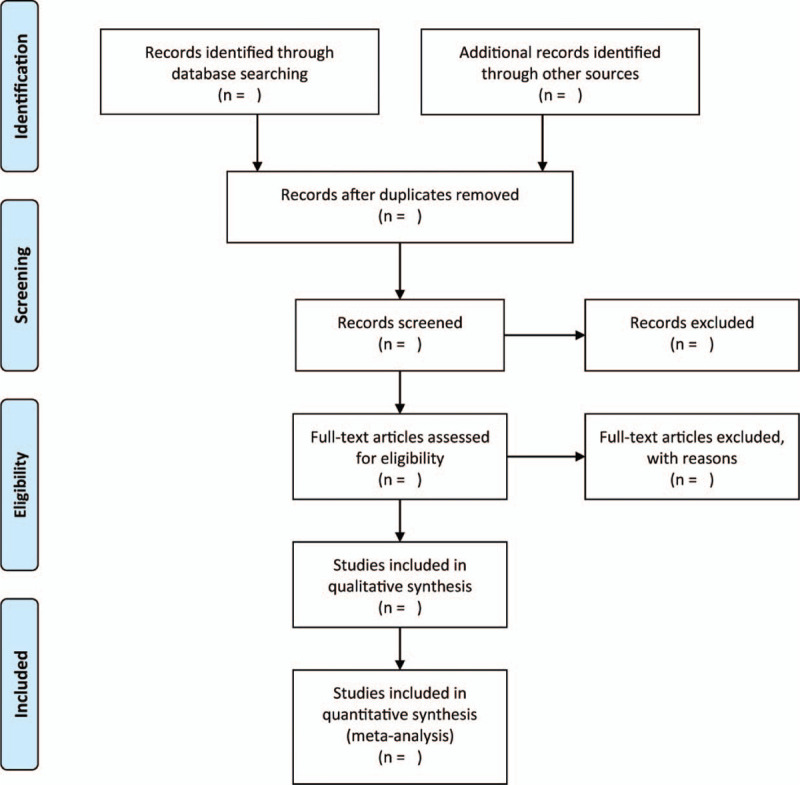
The research flowchart.

We will use 2 independent authors for screening the titles and abstracts extracted from the search. They will read potential articles and decide which are to consider for inclusion on the basis of prespecified inclusion criteria. Accordingly, if any disagreements on whether to include an article or not, the authors will resolve the disagreement through consensus. Figure 1 shows the detailed selection process.

### 6.2 Data extraction

Two independent authors will use a standardized form to extract all relevant data. The data extracted included demographics of participants, study methods, outcomes measures, and data required for diagnostic analysis (specific, sensitivity, and their 95% confidence interval). Any disagreements will be resolved by consensus.

### 6.3 Assessment of methodological quality

Two authors will independently employ the Quality Assessment of Diagnostic Accuracy Studies-2 tool to evaluate all literature's procedural or methodological quality in the study.^[14]^

### 6.4 Measures of treatment effect

Sensitivity and specificity will be used to assess the number of True/False and Negatives/Positives.

### 6.5 Dealing with missing data

The original authors will be contacted to verify the characteristics of studies or clarify missing or unclear outcome data.

### 6.6 Assessment of heterogeneity

The *I*^2^ statistic will be employed to measure heterogeneity. We will consider an *I*^2^ > 50% to provide evidence of statistic heterogeneity. We will also apply the random-effects model to merge data;^[15]^ otherwise, we will apply the fixed-effects model to merge data.^[16]^

### 6.7 Sensitivity analysis

Sensitivity analyses will be conducted where applicable to explore the sustainability of our findings.

## 7 Discussion

While numerous studies have reported the overall diagnostic value of serum HE-4 and CA-125 in patients experiencing OC, no systematic review has investigated the overall diagnostic accuracy of HE-4 and CA-125 among patients suffering from OC. We consider that our study is the first systematic review and a meta-analysis to investigate the overall diagnostic accuracy of HE-4 and CA-125 to diagnose patients suffering from OC. Therefore, our study could provide clinical evidence and represent a possibility as well as the future direction of OC diagnosis.

## Author contributions

**Conceptualization:** Hai-Ying Dai, Fang Hu.

**Data curation:** Fang Hu, Yuan Ding.

**Formal analysis:** Hai-Ying Dai, Fang Hu.

**Funding acquisition:** Yuan Ding.

**Investigation:** Hai-Ying Dai, Fang Hu.

**Methodology:** Hai-Ying Dai.

**Project administration:** Yuan Ding.

**Resources:** Hai-Ying Dai.

**Software:** Hai-Ying Dai, Fang Hu.

**Supervision:** Fang Hu, Yuan Ding.

**Validation:** Hai-Ying Dai.

**Visualization:** Hai-Ying Dai, Fang Hu, Yuan Ding.

**Writing – original draft:** Hai-Ying Dai, Fang Hu.

**Writing – review & editing:** Yuan Ding.
